# Clinical outcome comparison of percutaneous coronary intervention and bypass surgery in diabetic patients with coronary artery disease: a meta-analysis of randomized controlled trials and observational studies

**DOI:** 10.1186/s13098-019-0506-y

**Published:** 2019-12-19

**Authors:** ChuanNan Zhai, HongLiang Cong, Kai Hou, YueCheng Hu, JingXia Zhang, YingYi Zhang

**Affiliations:** 1 School of Medicine, NanKai University, Weijin Road No. 94, Nankai District, Tianjin, 300071 China; 2 Department of Cardiology, Tianjin Chest Hospital, Taierzhuang South Road No. 291, Jinnan District, Tianjin, 300350 China

**Keywords:** Diabetes mellitus, Coronary artery bypass surgery, Percutaneous coronary intervention, Meta-analysis

## Abstract

**Background:**

The optimal revascularization technique in diabetic patients with complex coronary artery disease (CAD), including left main CAD and multivessel coronary disease (MVD), remains controversial. The current study aimed to compare adverse clinical endpoints of coronary artery bypass graft (CABG) and percutaneous coronary intervention (PCI) in patients with diabetes mellitus (DM).

**Methods:**

Relevant studies were found from MEDLINE, OVID, Science Direct, Embase and the Cochrane Central database from January 2010 to April 2019. Risk ratio (RR) with 95% confidence interval (CI) was used to express the pooled effect on discontinuous variables. Outcomes evaluated were all-cause mortality, major adverse cardiac/cerebrovascular events (MACCE), cardiac death, myocardial infarction, stroke, and repeat revascularization.

**Results:**

Sixteen studies were included (18,224 patients). PCI was associated with the increase risk for MACCE (RR 1.59, 95% CI 1.38–1.85), cardiac death (RR 1.76, 95% CI 1.11–2.80), MI (RR 1.98, 95% CI 1.53–2.57), repeat revascularization (RR 2.61, 95% CI 2.08–3.29). The risks for all-cause mortality (RR 1.23, 95% CI 1.00–1.52) and stroke (RR 0.71, 95% CI 0.48–1.03) were similar between two strategies. Stratified analysis based on studies design and duration of follow-up showed largely similar findings with the overall analyses, except for a significant increased risk of all-cause mortality (RR 1.32, 95% CI 1.04–1.67) in long-term group, and CABG was associated with a higher stroke rate compared to PCI, which are results that were found in RCTs (RR 0.47, 95% CI 0.28–0.79) and mid-term groups (RR 0.39, 95% CI 0.23–0.66).

**Conclusions:**

CABG was superior to PCI for diabetic patients with complex CAD (including left main CAD and/or MVD), but might be associated with a higher risk of stroke mid-term follow-up.

*Number of Protocol registration* PROSPERO CRD 42019138505.

## Background

In recent years, the occurrence of diabetes mellitus (DM) in patients worldwide has been increasing rapidly [1]. The total number of patients with DM is expected to rise to nearly 600 million by 2035 [2]. As the critical risk factor for coronary artery disease (CAD), DM typically presents with diffuse comorbid atherosclerosis and multiple-vessel stenosis, which are poor prognostic indicators of revascularization strategies [3, 4]. Currently, coronary artery bypass grafting (CABG) has been recommended as the standard of care for patients with diabetes and complex anatomic diseases, including left main CAD [5]. However, with the application of drug-eluting stents (DESs) and the improvement of interventional technology, the incidence of restenosis and repeat revascularization after percutaneous coronary intervention (PCI) has been significantly reduced [6, 7]. PCI is regarded as an alternative to CABG, as it is less invasive, which is favored by more patients. Thus, a number of clinical studies have been conducted globally to estimate and compare the clinical effects and end-point outcomes of the two approaches in an effort to determine the best revascularization strategy for patients with DM and complex CAD [8–11].

Recently, two meta-analyses based on several published studies (e.g., Dai et al. [12]) found that the incidence of all-cause mortality (1–5 years follow-up) of DM patients underwent PCI did not differ significantly from those who underwent CABG. Similarly, Xin et al. [13] found no clear difference in mortality between CABG and PCI in patients with DM and serious coronary disease. However, these analyses assessed a limited number of studies. Furthermore, the evidence was not examined through stratified analysis of follow-up time and/or study type. These issues are important, as a recently published clinical trial [14] showed that the rate of mortality after PCI was significantly higher than CABG, differing from previous results.

Therefore, this study comprehensively examined research completed in the last 10 years, evaluating and comparing clinical outcomes of PCI or CABG in patients with complex coronary disease (including left main CAD and/or MVD) in an effort to determine the most appropriate revascularization strategy for patients with DM.

## Methods

This study was performed according to the Cochrane Collaboration guidelines and is reported in accordance with the Preferred Reporting Items for Systematic reviews and Meta-Analysis extension (PRISMA) statement [15, 16]. The protocol was registered in PROSPERO database (http://www.crd.york.ac.uk/PROSPERO/) under the number CRD42019138505.

### Inclusion criteria

Inclusion criteria were as follows: (1) types of studies: we included all randomized controlled trials (RCTs) and observational studies (OS); (2) types of participants: all patients with DM (including type 1 and 2 diabetes) included in studies were diagnosed with left main CAD, MVD, or both; (3) types of interventions: all patients underwent direct percutaneous coronary intervention (PCI) or coronary artery bypass grafting surgery (CABG); and (4) outcomes: the incidence of all-cause mortality of patients underwent PCI, comparison to patients with CABG. Other outcomes included the risk of cardiac death, MACCE, myocardial infarction (MI), stroke, or repeat revascularization. MACCE refers to major adverse cardiac events and cerebrovascular events, including death, MI, stroke or repeat revascularization. Subgroups analyses of the incidence of these endpoints were conducted according to different study designs (included RCTs and observational studies) and duration of follow-up (midterm: 1–3 years, long-term: > 3 years).

### Exclusion criteria

Exclusion criteria were the following: (1) overlapping and/or repetitive data; (2) review articles, single case reports, and noncomparable studies; (3) the number of diabetic patients for comparison was less than 50; (4) DESs were not used in interventional therapy; and (5) a follow-up period < 1 year.

### Search strategy

We searched for all relevant studies from MEDLINE (including PubMed), OVID, Science Direct, Embase and the Cochrane Central database, from January 2010 to April 2019. The following search terms were used to maximize search sensitivity and specificity: percutaneous coronary intervention, drug-eluting stents, coronary artery bypass graft, coronary bypass, left main coronary artery disease, multivessel disease, diabetes mellitus. Additionally, further relevant studies were identified through the reference list of review articles.

### Study selection

In the present study, two reviewers (CN Zhai and K Hou) independently screened the titles and abstracts of articles for eligibility criteria. Then, the full text of studies that potentially met inclusion criteria was inspected to determine which studies were included in analyses. If the two reviewers disagreed regarding the inclusions of a study, a consensus was reached by consulting a third researcher (HL Cong).

### Data extraction and quality assessment

Data from all included articles were extracted independently by two investigators (CN Zhai and K Hou). Data included the study title, publication date, authors, studies design, number of patients, coronary lesion, duration of follow-up, and the risk of every endpoint, etc. the corresponding authors of the included studies were contacted to obtain any required information that was missing. The total extracted data were verified by a third investigator (HL Cong). Three reviewers (CN Zhai, K Hou, and YY Zhang) independently evaluated the potential risk of bias of randomized trials by applying the Cochrane Collaboration’s tool [17] and the quality of observational studies by using the Newcastle–Ottawa Scale criteria [18].

### Statistical analysis

Data analysis was conducted using the RevMan software, version 5.1 (the Nordic Cochrane Centre, the Cochrane collaboration, Copenhagen, Denmark) and STATA 12.0 software (StataCorp, College Station, TX, USA). The risk ratios (RRs) and 95% confidence intervals (CIs) were used to evaluate the dichotomous outcomes, such as the incidence of all-cause mortality. To combine the separate statistics, the inverse variance and Mantel–Haenszel techniques were used. The heterogeneity was investigated by the use of the Q statistic, and *P* values < 0.05 was regarded as statistically significant, a random-effects model was used in the above circumstances. Sensitivity analysis was conducted using an exclusion method whereby multiple analyses were performed, with a different study excluded in each analysis of the clinical outcome.

Publication bias was evaluated statistically using Begg funnel plots and Egger’s bias test. The above methods measured the degree of funnel-plot asymmetry statistically [19, 20]. The Begg adjusted rank correlation test was used to evaluate the relationship between the test accuracy estimate and their variances. The deviation of Spearman ρ values from zero provided an estimate of the funnel-plot asymmetry. Positive values indicated a trend toward higher levels of test accuracy in studies with smaller sample sizes. The Egger bias test detects funnel-plot asymmetry by determining whether the intercept deviates significantly from zero in a regression of the standardized effect estimates against their precision values.

## Results

### Search results and characteristics of included studies

**Fig. 1:**
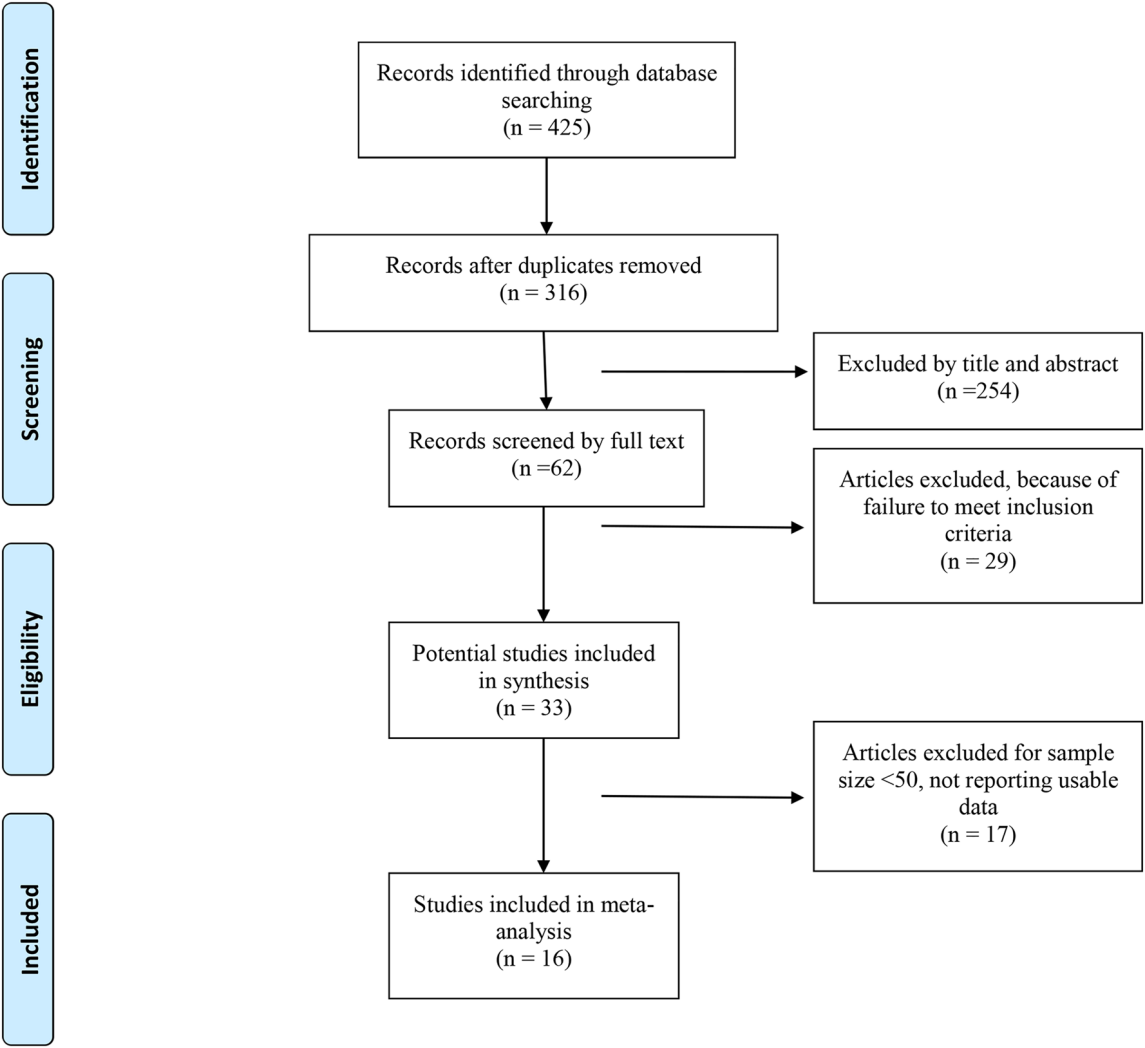
Flow of studies through the meta-analysis

A total of 425 articles were found during the initial electronic search, after removal of duplicate studies. After screening, 409 failed to meet eligibility criteria. Eventually, a total of 16 articles, which included 7 randomized controlled trails [5, 8, 9, 14, 21–23] and 9 observational studies [11, 24–31], met all eligibility criteria (Fig. 1).

**Fig. 2:**
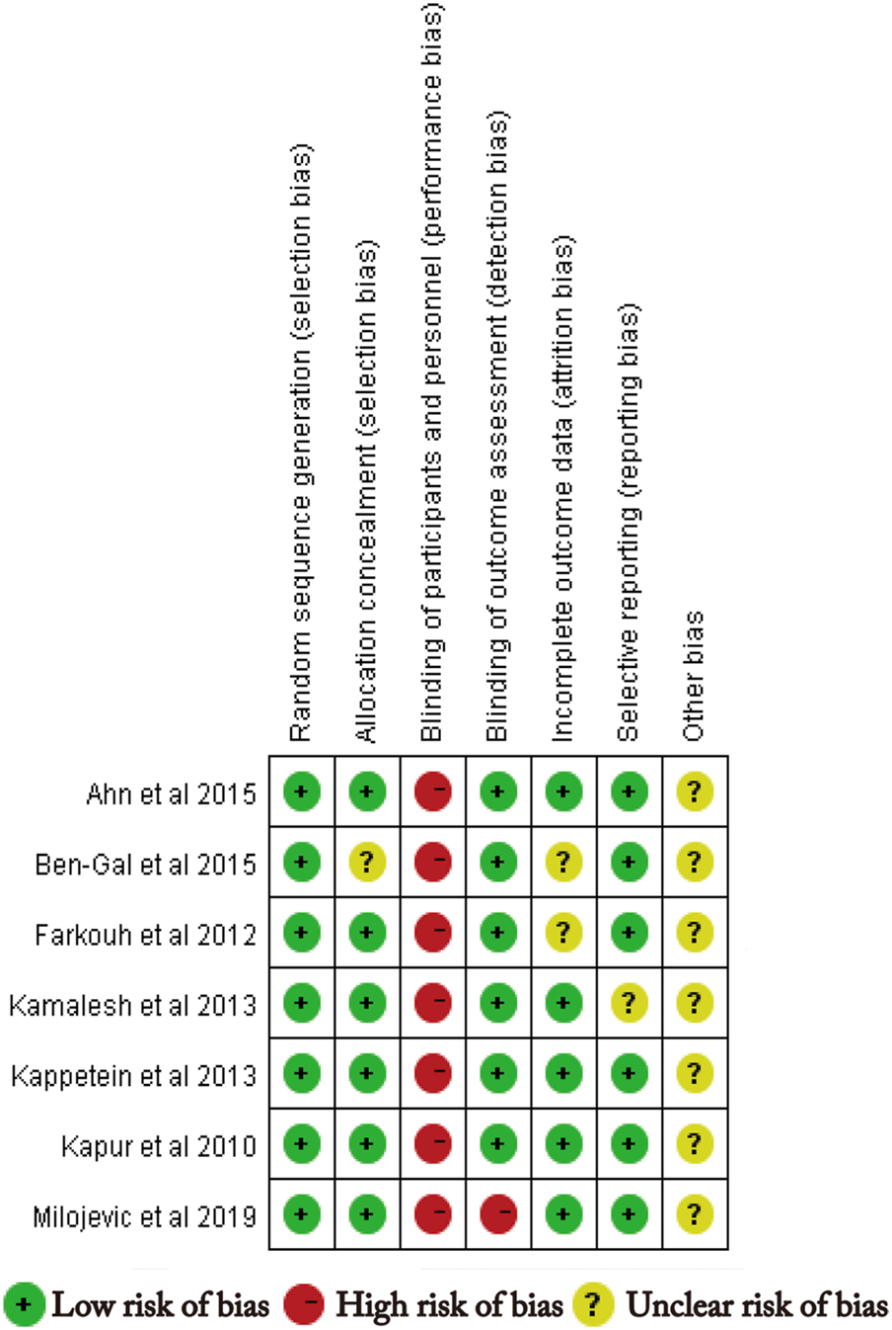
Methodological quality of included RCTs. This risk-of-bias tool incorporates assessment of randomization (sequence generation and allocation concealment), blinding (participants, personnel, and outcome assessors), completeness of outcome data, selection of outcomes reported, and other sources of bias. The items were scored with “yes,” “no,” or “unsure.”

**Fig. 3:**
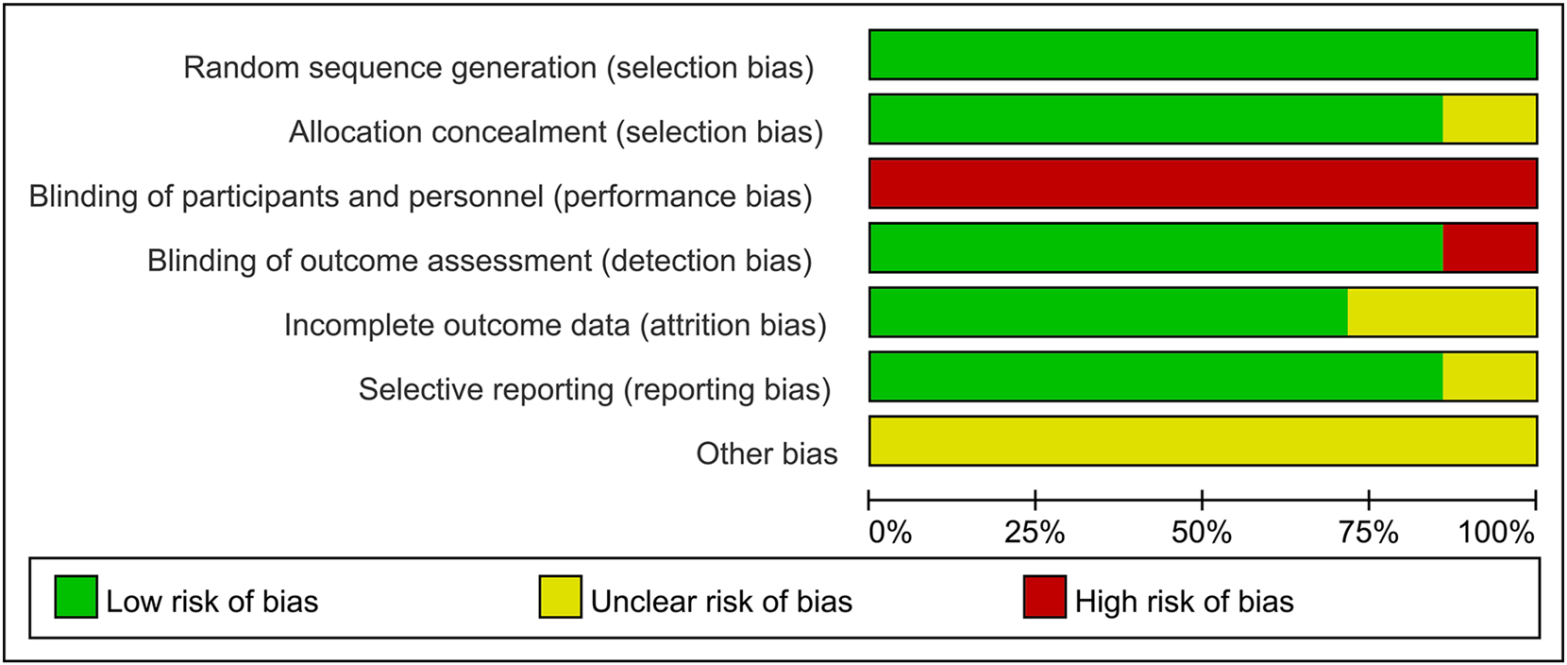
Risk of bias. Each risk-of-bias item is presented as percentages across included RCTs, which indicate the proportion of different levels of risk of bias for each item

A total of 18,224 patients with DM receiving PCI (*n* = 9863) and CABG (*n* = 8361) were included in the present analysis. Table 1 showed the general characteristics of the individuals included in the studies investigated. Some raw data of characteristics were not fully available. Although contact with the authors of the original studies was attempted, no responses were received. The methodological quality of included RCTs was presented in Fig. 2. Judgement about each risk of bias item are presented as percentages across RCTs in Fig. 3. Although the nature of the intervention made trials blinded for patients impossible, this was not considered a source of significant bias. The quality of observational studies is presented in Table 2 and they had high quality in their data outcome and clinical design.

**Table 1:** Characteristics of the included studies

Study	Year	Follow-up years	Samples P/C	Age, years P/C	Male, % P/C	HTN, % P/C	Smoke, % P/C	Dsl, % P/C	Stent type	Study design	Coronary lesion
Kapur et al. [21]	2010	1	172/178	64.3/63.6	70.7/77.9	20.8/18.7	24.6/23.2	NA	DES	RCT	MVD (2- or 3-vessel disease)
Luo et al. [24]	2012	3	99/127	65/66	70/98	72/87	43.0/54.0	15.0/31.0	DES	OS	Unprotected LM disease (and/or 1-, 2-, or 3-vessel coronary disease)
Farkouh et al. [5]	2012	1	953/947	63.2/63.1	73.2/69.5	NA	14.8/16.6	NA	SES or PES	RCT	MVD with stenosis of more than 70% in 2 or more major epicardial vessels
Kamalesh et al. [8]	2013	2	101/97	62.7/62.1	99.0/99.0	96.0/95.7	27.7/20.6	NA	DES	RCT	MVD (2- or 3-vessel disease)
Kappetein et al. [9]	2013	5	231/221	NA	NA	NA	NA	NA	PES	RCT	LM and/or 3-vessel disease
Ben-Gal et al. [23]	2015	1	1349/423	65.0/65.0	73.0/66.3	79.4/85.9	21.0/24.0	61.8/72.7	DES	RCT	LM and/or 2-, 3-vessel disease
Bangalore et al. [25, 34]	2015	4	773/773	64.9/64.7	68.0/68.0	NA	NA	NA	EES	OS	MVD (2-, 3-vessel disease and without LM)
Marui et al. [26]	2015	5	1065/933	68.7/67.8	68.0/73.0	88.0/84.0	25.0/25.0	NA	DES	OS	LM and/or 3-vessel disease
Ahn et al. [22]	2015	5	102/90	NA	NA	NA	NA	NA	SES	RCT	Unprotected LM disease with stenosis of more than 50%
Naito et al. [27]	2015	3.8	256/227	72.7/72.7	78.1/68.3	77.0/74.0	58.6/62.6	76.6/68.7	DES	OS	LM and/or 2-, 3-vessel disease
Yu et al. [28]	2015	7.1	143/131	65.0/66.0	72.0/77.9	68.5/65.6	49.7/45.8	53.8/37.4	DES	OS	Unprotected LM and 1-, 2-, or 3-vessel coronary disease
Zheng et al. [29]	2016	3	348/806	NA	NA	NA	NA	NA	DES	OS	LM and/or 1-, 2-, 3-vessel disease
Li et al. [30]	2017	10	406/406	41.9/42.0	366/369	60.8/63.5	66.0/65.3	NA	DES	OS	LM and/or 1-, 2-, 3-vessel disease
Ramanathan et al. [11]	2017	5	2710/1865	67.3/65.2	72.0/73.2	88.1/91.8	NA	77.5/79.5	DES	OS	MVD with stenosis of more than 70% in 2 or more major epicardial vessels
Nagendran et al. [31]	2018	5	869/869	65.1/65.1	23.0/21.0	83.0/85.0	18.0/20.0	80.0/82.0	DES	OS	LM and/or 2-, 3-vessel disease
Milojevic et al. [14]	2019	3	286/268	NA	NA	NA	NA	NA	EES	RCT	LM and/or 1-, 2-, or 3-vessel coronary disease

*HTN* hypertension, *Dsl* dyslipidemia, *P/C* percutaneous coronary intervention versus coronary artery bypass surgery, *DES* drug-eluting stents, but type of the stents is not available, *SES* sirolimus-eluting stent, *PES* paclitaxel-eluting stent, *EES* everolimus-eluting stent, *MVD* multivessel disease, *LM* left main, *RCT* randomized controlled trials, *OS* observational studies, *NA* not available

**Table 2:** Newcastle–Ottawa scale (NOS) for assessing quality of observational studies

Study	Selection				Comparability of the cohort	Outcome			Total score
	Representativeness of the exposed cohort	Selection of the nonexposed cohort	Ascertainment of exposure	Outcome not present at baseline		Assessment of outcome	Enough follow-up duration	Adequate follow-up	
Luo et al. [24]	*	*	*	*	**	*	*	*	9
Bangalore et al. [25, 34]	*	*	*	*	**	*	*	*	9
Marui et al. [26]	*	*	*	*	**	*	*	*	9
Naito et al. [27]	*	*	*	*	**	–	*	*	8
Yu et al. [28]	*	*	*	*	**	*	*	*	9
Zheng et al. [29]	*	*	*	*	**	*	*	*	9
Li et al. [30]	*	*	*	*	**	–	*	*	8
Ramanathan et al. [11]	*	*	*	*	**	*	*	*	9
Nagendran et al. [31]	*	*	*	*	**	*	*	*	9

The scale assigns 4 points for selection, 2 points for comparability and 3 points for outcome (* 1 point; ** 2 points). Score of 5 to 6 considered as moderate quality and 7 to 9 as high quality

### All-cause mortality

**Fig. 4:**
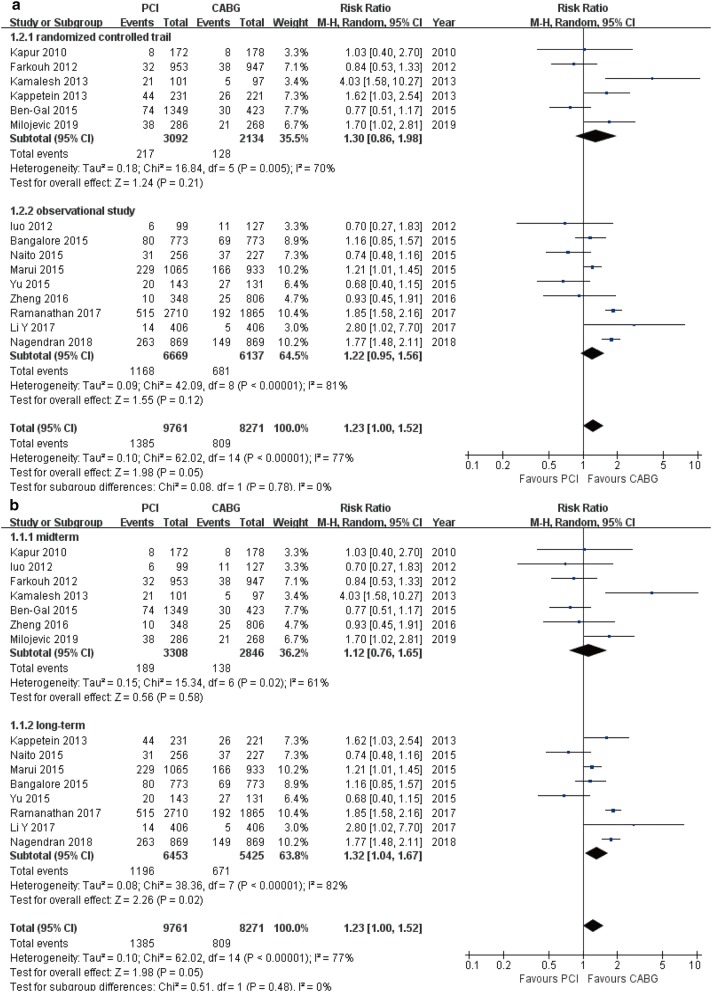
Forest plots for all-cause mortality between PCI and CABG patients (**a** subgroup analysis of study design; **b** subgroup analysis of follow-up periods)

All-cause mortality was reported in 15 studies (18,032 patients) (Table 3). Comparing with patients undergoing CABG, all-cause mortality was significantly higher in patients who received PCI (RR 1.23, 95% CI 1.00–1.52, *P* = 0.05). In analysis stratified by study design and duration of follow-up, consistent findings were observed in the subgroup of long-term follow-up (RR 1.32, 95% CI 1.04–1.67, *P* = 0.02). A trend toward increased risk was detected in the subgroups of RCTs (RR 1.30, 95% CI 0.86–1.98, *P* = 0.08), OS (RR 1.22, 95% CI 0.95–1.56, *P* = 0.12) and mid-term follow-up (RR 1.12, 95% CI 0.76–1.65, *P* = 0.58; Fig. 4a, b).

**Table 3:** Total meta-analysis outcomes and stratified analysis of each endpoint based on study design and duration of follow-up

Endpoints	Subgroup	Study, *n*	RR	95% CI	*P*_value_	*I*^2^ (%)	*P*_heterogeneity_
All-cause death	Total	15	*1.23*	*1.00–1.52*	*0.05*	77	< 0.001
	RCT	6	1.30	0.86–1.98	0.08	70	0.005
	OS	9	1.22	0.95–1.56	0.12	81	< 0.001
	Mid-term	7	1.12	0.76–1.65	0.58	61	0.02
	Long-term	8	*1.32*	*1.04–1.67*	*0.02*	82	< 0.001
MACCE	Total	8	*1.59*	*1.38–1.85*	*< 0.001*	68	0.003
	RCT	5	*1.40*	*1.20–1.63*	*< 0.001*	26	0.25
	OS	3	*1.87*	*1.72–2.03*	*< 0.001*	0	0.52
	Mid-term	3	*1.31*	*1.11–1.54*	*0.001*	14	0.31
	Long-term	5	*1.84*	*1.70–1.99*	*< 0.001*	0	0.58
Cardiac death	Total	7	*1.76*	*1.11–2.80*	*0.02*	71	0.002
	RCT	4	*2.25*	*1.28–3.98*	*0.005*	65	0.04
	OS	3	1.20	0.92–1.56	0.18	0	0.93
	Mid-term	4	1.99	0.96–4.15	0.07	70	0.02
	Long-term	3	1.37	0.99–1.90	0.06	18	0.30
MI	Total	11	*1.98*	*1.53–2.57*	*< 0.001*	64	0.002
	RCT	5	1.35	0.97–1.86	0.07	42	0.14
	OS	6	*2.44*	*2.07–2.88*	*< 0.001*	3	0.40
	Mid-term	5	1.53	0.95–2.48	0.08	69	0.01
	Long-term	6	*2.35*	*2.01–2.73*	*< 0.001*	0	0.61
Stroke	Total	12	0.71	0.48–1.03	0.07	66	< 0.001
	RCT	6	*0.44*	*0.27–0.71*	*< 0.001*	0	0.76
	OS	6	0.95	0.62–1.45	0.81	72	0.003
	Mid-term	6	*0.39*	*0.23–0.66*	*< 0.001*	0	0.86
	Long-term	6	0.95	0.64–1.41	0.79	72	0.004
Repeat revascularization	Total	12	*2.61*	*2.08–3.29*	*< 0.001*	79	< 0.001
	RCT	5	*2.22*	*1.46–3.39*	*< 0.001*	78	0.001
	OS	7	*2.92*	*2.21–3.87*	*< 0.001*	79	< 0.001
	Mid-term	6	*2.88*	*1.66–4.99*	*< 0.001*	84	< 0.001
	Long-term	6	*2.56*	*2.02–3.24*	*< 0.001*	75	0.001

Italic values indicate significance of *P* value (*P*_value_ and *P*_heterogeneity_ < 0.05)

*RR* risk ratio, *CI* confidence intervals, *MACCE* major adverse cardiac and cerebrovascular event, *MI* myocardial infarction, *RCT* randomized controlled trials, *OS* observational studies, *Mid-term* 1–3 years follow-up, *Long-term* > 3 years follow-up

### Macce

**Fig. 5:**
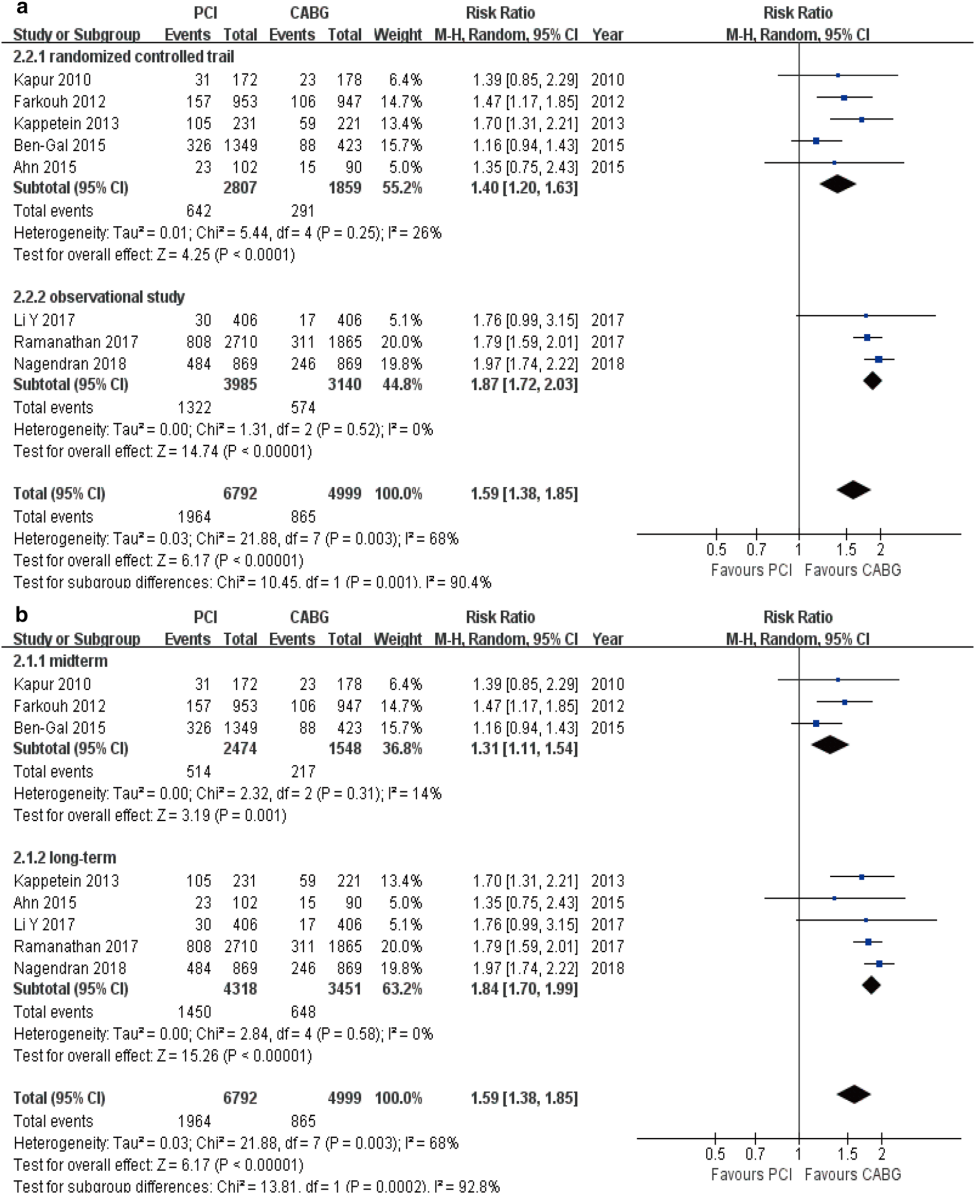
Forest plots for MACCE between PCI and CABG patients (**a** subgroup analysis of study design; **b** subgroup analysis of follow-up periods)

The overall incidence of MACCE was higher in the PCI group compared with CABG group (8 studies; 11,791 patients; RR 1.59, 95% CI 1.38–1.85, *P* < 0.001) (Table 3). The same statistically significant differences were found in stratified analyses [RCTs (RR 1.40, 95% CI 1.20–1.63, *P* < 0.001); OS (RR 1.87, 95% CI 1.72–2.03, *P* < 0.001); mid-term follow-up (RR 1.31, 95% CI 1.11–1.54, *P* = 0.001); and long-term follow-up (RR 1.84, 95% CI 1.70–1.99, *P* < 0.001); Fig. 5a, b].

### Cardiac death

**Fig. 6:**
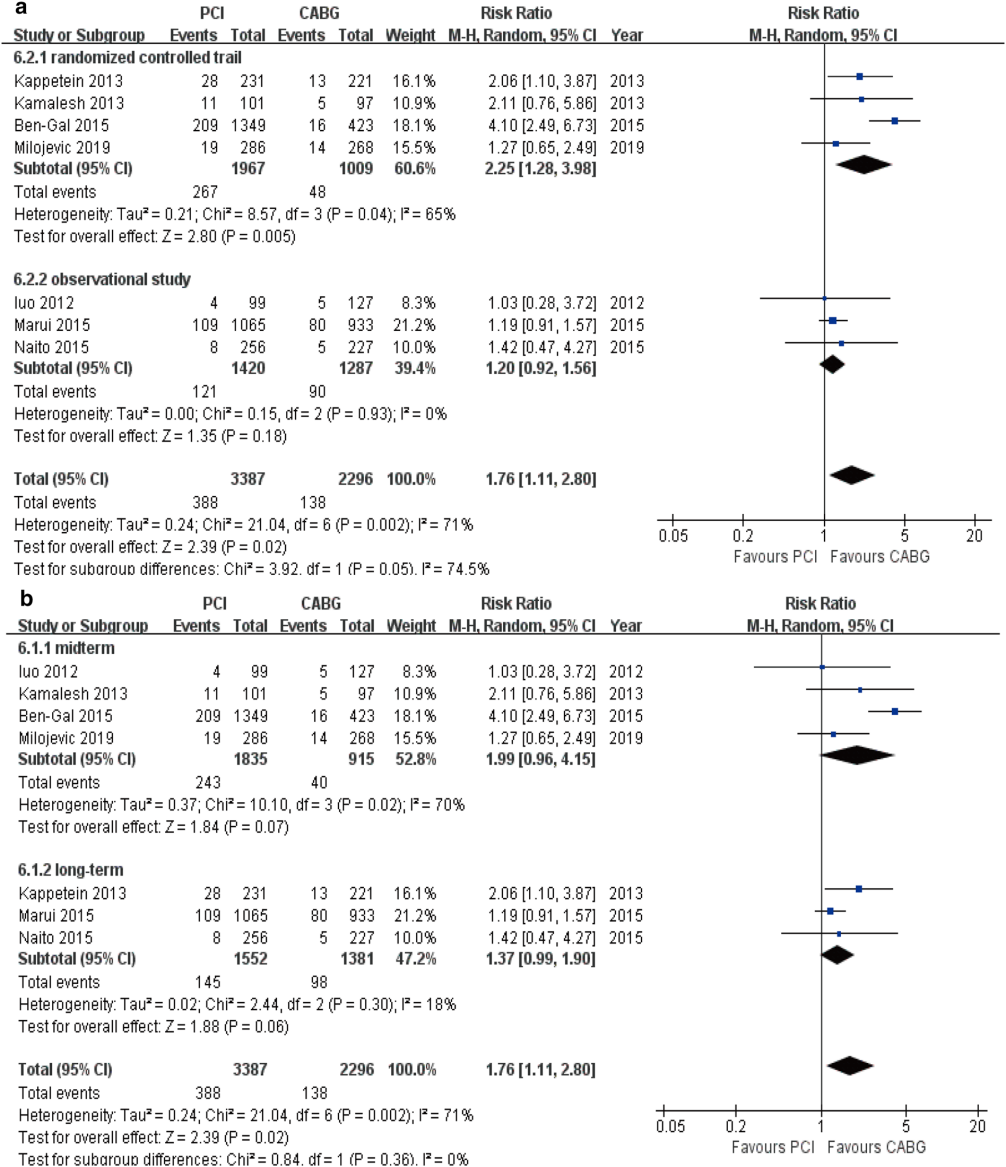
Forest plots for cardiac death between PCI and CABG patients (**a** subgroup analysis of study design; **b** subgroup analysis of follow-up periods)

Compared to the CABG group, patients receiving PCI demonstrated a higher risk of cardiac death (7 studies; 5683 patients; RR 1.76, 95% CI 1.11–2.80, *P* = 0.02) (Table 3). This was seen in the RCTs subgroup (RR 2.25, 95% CI 1.28–3.98, *P* = 0.005). In other stratified analyses, although there was no statistically significant findings, the similar trend toward increased risk was observed in OS (RR 1.20, 95% CI 0.92–1.56, *P* = 0.18), mid-term follow-up (RR 1.99, 95% CI 0.96–4.15, *P* = 0.07), and long-term follow-up (RR 1.37, 95% CI 0.99–1.90, *P* = 0.06) subgroups (Fig. 6a, b).

### Myocardial infarction

**Fig. 7:**
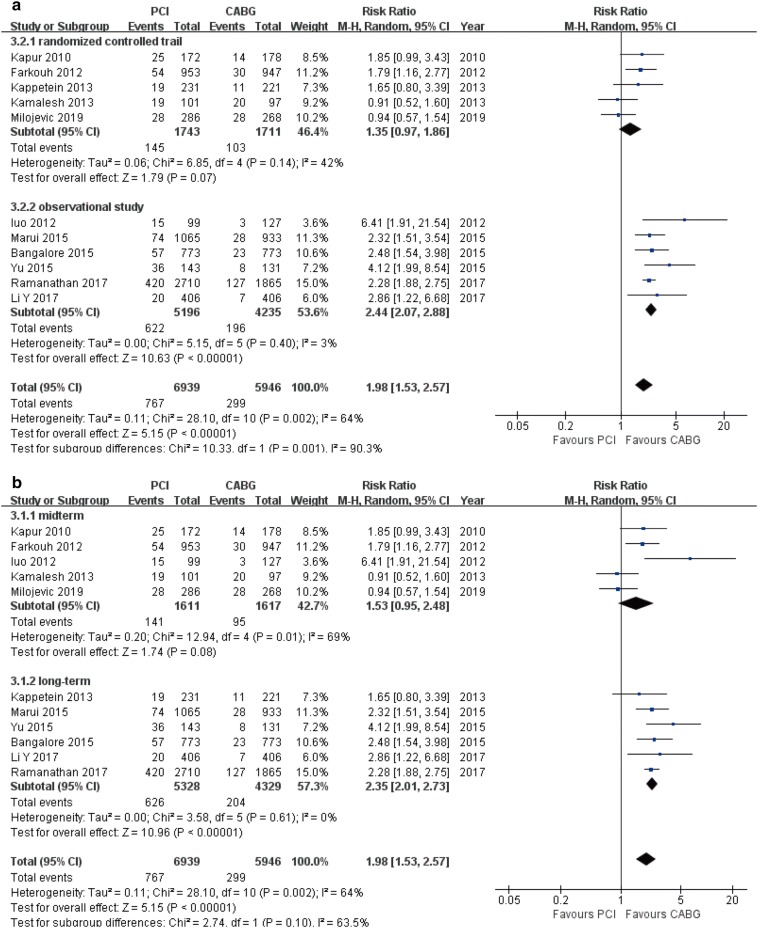
Forest plots for myocardial infarction between PCI and CABG patients (**a** subgroup analysis of study design; **b** subgroup analysis of follow-up periods)

There was a statistically significant increase in the risk of myocardial infarction in patients undergoing PCI compared to patients undergoing CABG (11 studies, 12,885 patients, RR 1.98, 95% CI 1.53–2.57, *P* < 0.001) (Table 3). This result was consistent in OS (RR 2.44, 95% CI 2.07–2.88, *P* < 0.001) and long-term follow-up (RR 2.35, 95% CI 2.01–2.73, *P* < 0.001) subgroups. A trend toward increased risk was observed in RCTs (RR 1.35, 95% CI 0.97–1.86, *P* = 0.07) and mid-term follow-up subgroups (RR 1.53, 95% CI 0.95–2.48, *P* = 0.08; Fig. 7a, b).

### Stroke

**Fig. 8:**
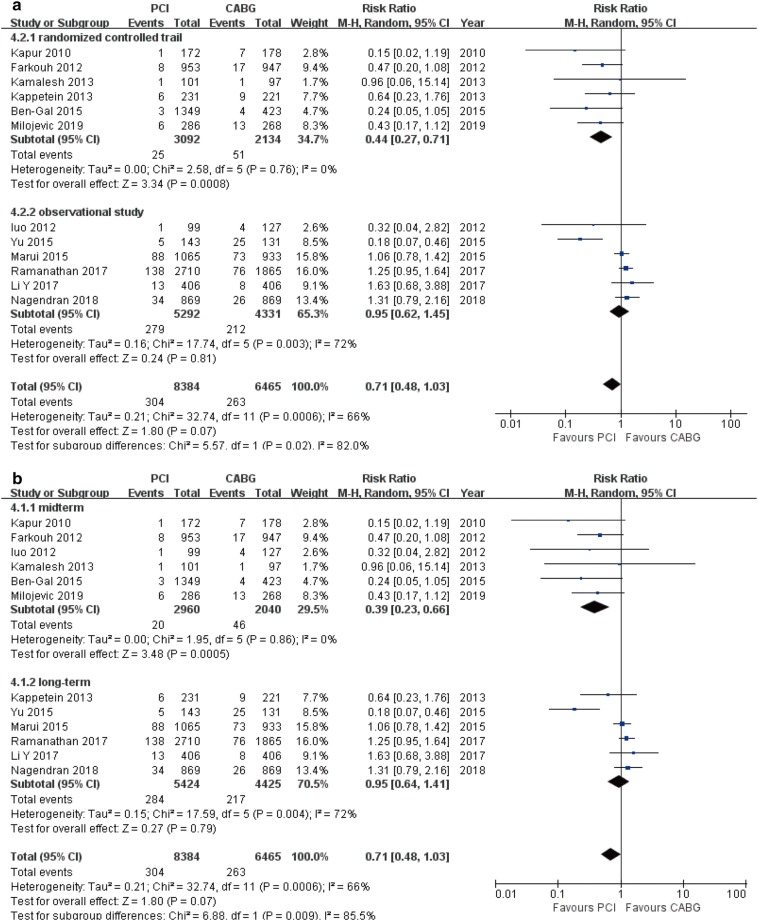
Forest plots for stroke between PCI and CABG patients (**a** subgroup analysis of study design; **b** subgroup analysis of follow-up periods)

There showed no significant difference in incidence of stroke between PCI and CABG (12 studies; 14,849 patients; RR 0.71, 95% CI 0.48–1.03, *P* = 0.07) (Table 3). Analysis by study design and follow-up time found that the rate of stroke was lower in the PCI patients compared with CABG patients in RCTs (RR 0.44, 95% CI 0.27–0.71, *P* < 0.001) and mid-term follow-up subgroups (RR 0.39, 95% CI 0.23–0.66, *P* < 0.001). The risk of stroke was similar in both OS (RR 0.95, 95% CI 0.62–1.45, *P* = 0.81) and long-term follow-up subgroups (RR 0.95, 95% CI 0.64–1.41, *P* = 0.79; Fig. 8a, b).

### Repeat revascularization

**Fig. 9:**
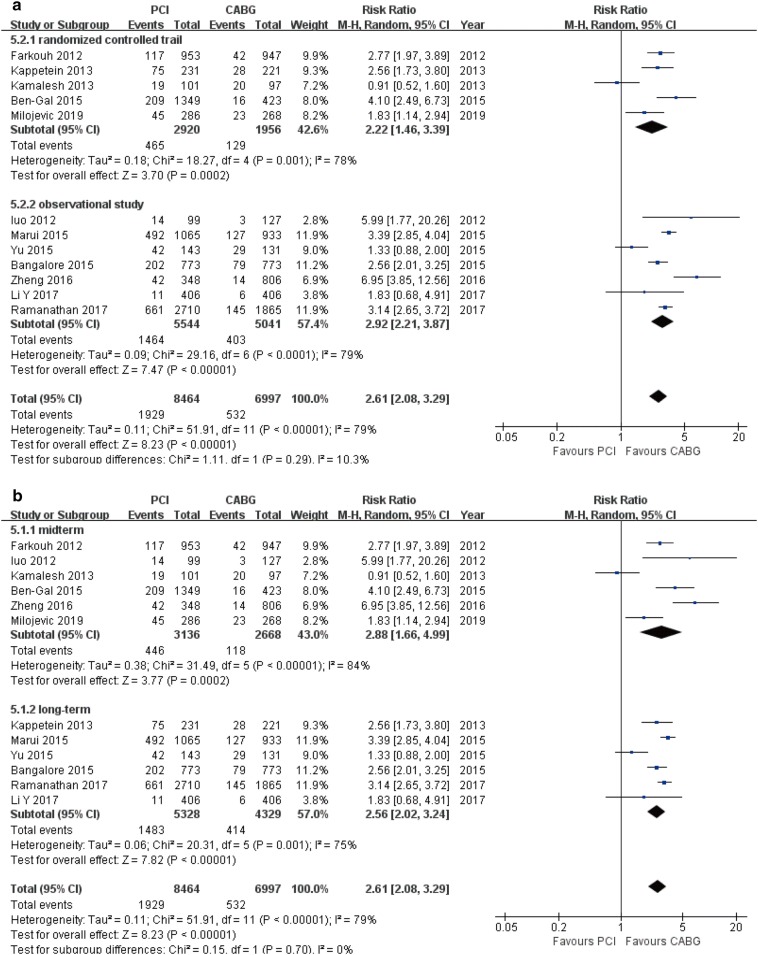
Forest plots for repeat revascularization between PCI and CABG patients (**a** subgroup analysis of study design; **b** subgroup analysis of follow-up periods)

Overall, there was a statistically significant increase in the risk of repeat revascularization in patients undergoing PCI compared with CABG (12 studies; 15,461 patients; RR 2.61, 95% CI 2.08–3.29, *P* < 0.001) (Table 3). This effect was also demonstrated in all multiple stratified analyses [RCTs (RR 2.22, 95% CI 1.46–3.39, *P* < 0.001); OS (RR 2.92, 95% CI 2.21–3.87, *P* < 0.001); mid-term follow-up (RR 2.88, 95% CI 1.66–4.99, *P* < 0.001); and long-term follow-up (RR 2.56, 95% CI 2.02–3.24, *P* < 0.001); Fig. 9a, b].

### Sensitivity analyses and publication bias

Publication bias tests were performed for all endpoints of included studies. There was no evidence of publication bias (Table 4). Sensitivity analyses in overall endpoints showed that the results of these analyses were not excessively influenced by any of the included studies.

**Table 4:** Publication bias assessment of this meta-analysis

Endpoints	Egger’s test		Begg’s test	
	t-value	*p*	t-value	*p*
All-cause mortality	− 1.39	0.189	0.40	0.692
Cardiac death	0.56	0.597	0.30	0.764
MACCE	− 1.68	0.144	0.37	0.711
MI	− 0.14	0.889	0.62	0.533
Stroke	− 2.06	0.066	0.89	0.373
Repeat revascularization	− 0.89	0.395	0.34	0.732

*MACCE* major adverse cardiac and cerebrovascular event, *MI* myocardial infarction

## Discussion

The optimal revascularization strategy for patients with DM and complex CAD, including left main CAD, is an important issue for cardiovascular experts. In clinical practice, PCI is more acceptable to patients with DM because of less trauma and faster recovery. CABG is more invasive than PCI and has a higher risk of adverse cerebral vascular events during perioperative period [32]. Although PCI is more likely to be associated with higher rate of adverse events after revascularization, previous studies [13] have reported no difference in mortality between PCI and CABG and CABG was associated with excess stroke [33, 34], making it difficult for physicians to assess the two strategies. This study pooled data from 16 studies, which included 18,224 diabetic patients (follow-up ≥ 1 year) with left main CAD and/or MVD, undergoing either PCI or CABG. At the long-term follow-up (> 3 years), we found that PCI was significantly associated with a high risk of all-cause mortality, MACCE, MI, repeat revascularization, although the risk of stroke was lower at the midterm follow-up (1–3 years). CABG was significantly associated with a lower risk of long-term mortality and other adverse clinical endpoints compared to PCI in patients with DM.

Previous studies have compared the two revascularization strategies and found different results than those observed in the current study. Although no separate analysis of diabetic cohorts was performed, a meta-analysis by Zhang et al. [35] suggested that PCI, with newer generation DES, might be a safe alternative revascularization strategy for left main CAD. However, it was noted that this method presented a higher risk of repeat revascularization. Mahmoud et al. [36] compared the clinical outcomes between these two strategies found that PCI was associated with a lower early risk of MACCE, while the risk of all-cause mortality, MI, and stroke were similar to CABG at long-term follow-up. Additionally, an in-depth comparative study of patients with DM [12] found that the risk of mortality (1–5 year follow-up) was not significantly different between PCI and CABG patients with DM; results that were supported by a recent study found no obvious difference in the incidence of all-cause mortality between the two strategies [13].

While the above-mentioned studies differ from the current results, several other studies have reported results similar to those reported here. For example, a study by Bundhun et al. [33], which involved 1297 patients with insulin-treated type 2 DM, found that, compared to PCI, CABG was associated with lower risk of several adverse long-term clinical outcomes, including mortality. However, it was also found that the rate of stroke was higher in patients who received CABG. A meta-analysis by Lee et al. [37] found a lower rate of mortality and MACCE in patients who underwent CABG. These authors also pointed out that insulin dependence had no influence on the clinical endpoints between the two therapy strategies. Smit et al. [38] observed that CABG was associated with a significantly lower risk of mortality and repeat revascularization in patients with DM or MVD, but that stroke was more common after this procedure. However, it should be noted that the studies included in this meta-analysis had relatively few patients with DM.

A previous study [24] identified that there was a prognostic impact of DM on treatment effects by either PCI or CABG, and that, in terms of both safety and efficacy, PCI was inferior to CABG in the diabetic group. CABG is recommended as a more appropriate revascularization strategy in patients with DM and complex coronary lesions. A high risk of restenosis was observed in patients with DM who underwent PCI, which may be associated with the need for more than two stents in MVD, as well as the continuous progression of diffuse atherosclerosis in non-culprit vessels [39]. At present, it is recommended to apply SYNTAX scores to the selection of revascularization strategies [40, 41]. Although the SYNTAX score might be considered useful in interventional cardiology, it has some limitations [42]. Compared with PCI, there was a higher risk of periprocedural stroke in patients undergoing CABG [43]. Moreover, post-operative problems or complications, such excessive sedation, ventilation, the use of intra-aortic balloon pump to help the heart, administration of inotropes, wound infection, hemorrhage, and pneumonia in patients with DM, were also elevated than those in non-diabetic patients [44, 45] and needed to be monitored and reasonably prevented.

Several studies have identified that adverse events were related to the severity of diabetes; for example, glycosylated hemoglobin (HbA1c) level and insulin therapy were independent risk factors for the development of post-surgery complications [46, 47]. Moreover, it is well-known that other risk factors such plasma homocysteine (Hcy) and c-reactive protein (CRP) have also an important role in CVD [48, 49], and it is a modifiable risk factors for restenosis [50]. Intervention for these risk factors and optimal glycemic control are the critical component of diabetes management. A recent study demonstrated that the use of sodium glucose cotransporter 2 inhibitors (SGLT2-is) reduces the risk of major cardiovascular events [51, 52]. In addition, the abnormal platelet activation observed in patients with DM is conducive to the formation of pathological thrombosis and the progression of cardiovascular diseases [53]. Therefore, improve interventional techniques, optimize antiplatelet/glycemic management, and the reduction of long-term adverse prognosis require further research.

In addition, despite subgroup analysis, heterogeneity still exited. We deemed that several clinical heterogeneity could not be eliminated. Among possible reasons for heterogeneity, differences in surgical techniques and differences in procedures of percutaneous transluminal coronary angioplasty (PTCA) (e.g., type of stent, concomitant medication, preparation for the prevention of contrast-induced nephropathy) could account for diversities in results across studies. Furthermore, heterogeneity may also have been caused by study design. Therefore, because of limited information obtained from original studies, heterogeneity cannot be completely resolved. Accordingly, although the results of present meta-analysis should be considered appropriately, methodological quality defects and clinical heterogeneity should be considered when interpreting the findings.

The number of published large-scale studies on patients with DM is currently small. To the best of our knowledge, the current study has the largest sample size of patients left main CAD and/or MVD to date. In addition, the current study compared the mid-term and long-term clinical outcomes between PCI and CABG. We found that CABG is superior to PCI in patients with DM, especially in differences of MACCE and repeat revascularization in all multiple stratified analyses. Although, it should be noted that the risk of stroke in CABG patients was higher than PCI patients at the mid-term follow-up, no statistically significant difference was observed at long-term follow-up. Our meta-analysis has higher accuracy, reliability, and statistical power due to the amplified sample size, resulting from the combination of the original studies. Thus, the current results could be considered stronger evidence than any one individual study. We hope that these results will assist diabetics and physicians in choosing the most appropriate revascularization strategy.

### Limitations

Some limitations of this meta-analysis should be considered: (1) because of the limited data available, this study was unable to conduct more in-depth stratified analysis based on the complex lesions of the coronary artery in diabetic patients; (2) we could not conduct a subgroup analysis comparing different type of DES (drug-eluting stent) and in-depth stratification on the basis of diabetic patients’ different drugs or comorbidity, due to limited studies and inadequate access to data; (3) the results of the included observational studies may have been selectively reported; and (4) due to the incomplete demographic data of diabetes patients, we cannot evaluate for heterogeneity by patient-level covariates. Unfortunately, heterogeneity of these variables (e.g., surgical experience, interference of diabetic complications, differences in drugs strategies) can never be completely resolved.

## Conclusions

CABG was superior to PCI in patients with DM and complex CAD (including left main CAD and/or MVD) regard to all-cause mortality, MACCE, MI, repeat revascularization, but is associated with a higher risk of stroke at mid-term follow-up. Further rigorous, high-quality research is required to confirm these conclusions, however, due to the several limitations of the current studies.

## Data Availability

Datasets are available through the corresponding author upon reasonable request.

## Abbreviations

CAD: coronary artery disease
MVD: multivessel coronary disease
CABG: coronary artery bypass graft
PCI: percutaneous coronary intervention
DM: diabetes mellitus
RR: risk ratio
CI: confidence interval
MACCE: major adverse cardiac/cerebrovascular events
MI: myocardial infarction
RCTs: randomized controlled trials
OS: observational studies
DESs: drug-eluting stent
HbA1c: glycosylated hemoglobin
Hcy: homocysteine
CRP: c-reactive protein
SGLT2-is: sodium glucose cotransporter 2 inhibitors
